# The knowledge and use of population-based methods for caries detection

**DOI:** 10.1186/s12903-018-0612-5

**Published:** 2018-08-29

**Authors:** Ana Luiza Sarno Castro, Maria Isabel Pereira Vianna, Carlos Maurício Cardeal Mendes

**Affiliations:** 1 Department of Health, State University of Feira de Santana, Transnordestina, s/n, Novo Horizonte, Feira de Santana, Bahia CEP 44036-900 Brazil; 2 Department of Public Oral Health, School of Dentistry, Federal University of Bahia, Araújo Pinho, 62, Canela, Salvador, Bahia CEP 40110040 Brazil; 3 Postgraduate Studies in Interactive Processes of Organs and Systems, Health Science Institute, Federal University of Bahia, Avenida Reitor Miguel Calmon, 1272, Salvador, Bahia CEP 40231300 Brazil

**Keywords:** Epidemiology, DMF, New indexes, Use, Knowledge, Surveys, Oral health

## Abstract

**Background:**

Since the 1980s, a wide variety of methods have been proposed to measure dental caries in the population, demonstrating a lack of consensus regarding the procedure that should be used for this purpose. The current study investigated the methods that are known and used by public oral health researchers and professors as well as the reasons that lead to the choice of a particular method.

**Method:**

In the context of an interview, a questionnaire was administered to public oral health researchers and professors who used caries indices and worked in Salvador and Feira de Santana, Bahia, Brazil from 2005 to 2015. A quantitative and descriptive approach was applied that adopted the multiple correspondence analysis (MCA) technique to assess the associations among responses.

**Results:**

The decayed, missing, and filled index (DMF) was the only measurement known by all respondents, and although 45 of the 47 professors/researchers were dissatisfied with this index, only six had used other methods. This index was chosen because of its comparability and ease of application. The MCA revealed response associations among older, male participants who graduated from the Federal University of Bahia (UFBA) and who continued to use this index because of its comparability and because it is the index recommended by the World Health Organization (WHO) and the Brazilian Ministry of Health (MS). Another group was also observed that consisted of younger females who graduated from the State University of Feira de Santana (UEFS) or another university and who used the DMF because it is well-known, simple, and easy to apply.

**Conclusions:**

The DMF index was the most known and used method. Many respondents demonstrated a desire for change and were critical of the DMF; however, they did not know of and had not used newer methods for measuring dental caries. Greater importance should be placed on the problem of dental caries assessment in the population.

**Electronic supplementary material:**

The online version of this article (10.1186/s12903-018-0612-5) contains supplementary material, which is available to authorized users.

## Background

The use of effective methods to assess caries in a population determines the quality of information obtained from epidemiological surveys, which in turn affects the diagnostic accuracy of this condition and is the basis for the planning, monitoring, and assessment of oral health prevention and disease control actions [1].

Several methods are used to measure dental caries in the population. The most widely used index is the decayed, missing, and filled (DMF) assessment described by Klein and Palmer in 1937 [2]. However, this index was created before the decrease in the incidence of caries and the advances in cariology that have occurred over the past decades that emphasises the importance of early diagnosis and early treatment for initial caries lesions. For these reasons, the DMF does not include non-cavitated enamel caries lesions among its components [3].

The use of the DMF has been questioned because of its limitations [3]. Since the 1980s, several authors have proposed different methods to assess caries lesions, such as the NYVAD System [4], the Significant Caries Index (SIC) [5], the Sound-Equivalent Teeth (T-Health) [6], the Filled and Sound Teeth (FS-T) [6], the Reversible Dental Caries Index (IRCD; *Índice Reversível de Cárie Dental*), the Caries Activity Index (IAC; *Índice de Atividade de Cárie*) [7], and many others [8–10]. Among the new methods used to assess caries in a population is the International Caries Detection and Assessment System (ICDAS) [11] and the Caries Assessment Spectrum and Treatment (CAST) [12], which have both been internationally validated by several studies [13–15].

The number of methods proposed in recent decades demonstrates that the diagnosis of caries in a population is an important topic and that no consensus exists among researchers with regard to the most appropriate method for making a diagnosis [3]. As such, the current study aimed to identify the caries assessment systems that are known among public oral health researchers and professors, what methods they currently use, and the reasons behind their method of choice.

## Methods

The present study employed an exploratory cross-sectional opinion poll with convenience sampling (Additional file 1). Through an interview, a semi-structured questionnaire was developed by the authors and administered to public oral health researchers and professors who used caries indices in Salvador and Feira de Santana, Bahia, Brazil, from 2005 to 2015.

The regions of Feira de Santana and Salvador were chosen for this study because they contain 43.8% of the dental schools in Bahia (14 of the 32 dental schools that existed in Bahia at the time of the interview) [16], and Salvador is among the 10 cities in Brazil with the most specialists registered in the Federal Council of Dentistry of Brazil [17].

Two search procedures were conducted to identify the study population: First, professors who teach public oral health at dental institutions in Salvador and Feira de Santana were identified; second, a search was performed using the PubMed, Lilacs, SciELO, and Google Scholar databases for researchers who had published articles describing their use of caries assessment methods during the stated period. Ultimately, 50 individuals met the study’s eligibility criteria.

The respondents formalised their acceptance by signing informed consent documents. This study was approved by the Research Ethics Committee of the Sciences Institute of the Federal University of Bahia, under CAAE number 48500115.2.0000.5662.

Instrument pre-testing was conducted with six dental professors who were subsequently excluded from the sample. This pre-test was performed from October 10 to 15, 2015, and the interviews were conducted from October 16, 2015 to March 8, 2016. The principal researcher conducted face-to-face interviews at the universities, offices, or houses of the interviewees based on their preference. The interviews lasted an average of 20 min and were recorded and then transcribed. The data were entered into the EPIDATA program and analysed using R statistical software [18].

A quantitative and descriptive analysis was conducted. The multivariate analysis technique known as a multiple correspondence analysis (MCA) was used. This tool allows for a set of categorical variables to be assessed based on both their intensity and degree of association [19].

## Results

Of the 50 individuals who were identified based on the search procedures, 47 agreed to participate. Their mean age was 46 years, with a standard deviation of 8 years. The mean time since graduation was 22 years, with a standard deviation of 8 years. Most participants were female (70.2%). Moreover, 28 individuals had graduated from UFBA (59.6%), 13 from UEFS (27.7%), and six (12.7%) from other universities (see Table 1).

**Table 1:** The distribution of professors and researchers by their personal characteristics, academic training, and place of work

VARIABLES	Number	Percent
SEX		
Female	13	70.2
Male	14	29.8
AGE		
Between 30 and 50 years	35	75.4
Between 51 and 70 years	12	24.6
TIME SINCE GRADUATION		
Between 8 and 17 years	12	24.6
Between 18 and 47 years	35	75.4
PLACE OF GRADUATION		
UFBA	28	59.6
UEFS	13	27.7
Other universities	6	12.7
PLACE OF WORK ^a^		
UEFS	19	40.4
UFBA	18	38.3
Other universities	10	21.3
Municipal or state health agency	8	17.0
Private practice	5	10.6

^a^The total number of responses is greater than the number of respondents because some respondents worked at more than one institution

Of the interviewees, 39 (83%) were public oral health professors, and 27 (57.4%) reported having performed research using caries assessment methods between 2005 and 2015. Of the individuals who were not university professors, five (10.6%) worked in the private sector (in offices), and three (6.4%) worked in the public sector (i.e., state organisations).

With regard to the specialties of the interviewees, 23 (48.9%) had postgraduate degrees in public health, three in health management (6.4%), and six (12.8%) in teaching methodology. The remaining 15 (32%) had specialties in other areas.

Table 1 shows that most professors worked at UEFS (40.4%) or UFBA (38.3%); the other 10 respondents (21.3%) worked at six different private universities or at the State University of Bahia (UNEB).

**Fig. 1:**
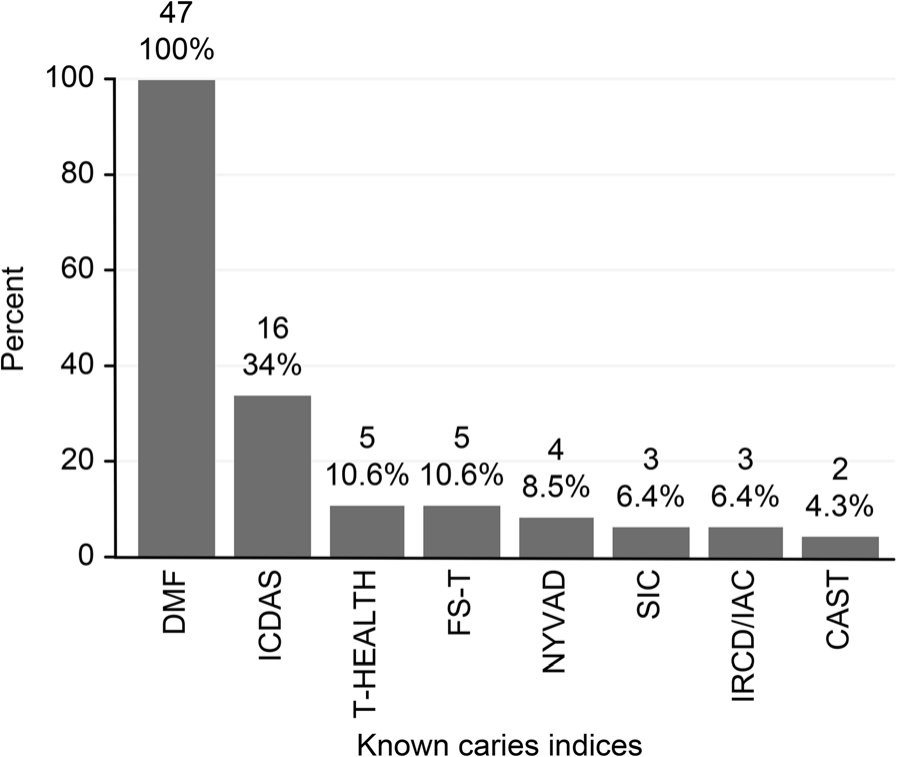
Knowledge of caries detection methods among public oral health professors and researchers

In response to the question about the indices that they knew, all respondents reported that they knew of the DMF and DMF with other indices, whereas 25 individuals (53.19%) knew of only the DMF. In addition, 22 respondents mentioned other indices (46.81%); of these participants, 16 (34%) knew of the ICDAS, five (10.6%) recalled the T-Health and FS-T, four (8.5%) mentioned the NYVAD, three (6.4%) recalled the SIC, IRCD, and IAC, and two (4.3%) knew the CAST (see Fig. 1 and Table 2).

**Table 2:** The distribution of professors and researchers by knowledge and use of indices

VARIABLES	Number	Percent
KNOWN INDICES		
DMF and DMF with other indices	47	100.0
Only DMF	25	53.2
DMF index and other indices	22	46.8
USED INDICES		
DMF and DMF with other indices	47	100.0
Only DMF	41	87.2
DMF index and other indices	06	12.8
INDICES LEARNED IN THE DEGREE PROGRAMME		
DMF and DMF with other indices	47	100.0
Only DMF	46	97.9
DMF index and other indices	01	2.1
INDICES TAUGHT IN LECTURES		
DMF and DMF with other indices	39^a^	100.0
Only DMF	34	87.2
DMF index and other indices	05	2.8
INDICES TAUGHT IN PRACTICAL CLASSES		
DMF and DMF with other indices	39^a^	100.0
Only DMF	37	94.9
DMF index and other indices	02	5.1

^a^ The total number of public oral health professors was 39 individuals

All of the professors taught the DMF index in their theory- (lectures) and practice-based classes. In addition to the DMF, three lectured on the ICDAS, and two addressed prevalence and incidence density measures. In practical classes, only one professor used the ICDAS, and one applied the prevalence coefficient (see Table 2).

According to Table 2, all respondents reported having had lectures and practical classes on the DMF during their degree programme. Only one respondent claimed to have learned about a different index during their programme. Most taught only the DMF in lectures (87.2%) and practical classes (94.9%).

**Fig. 2:**
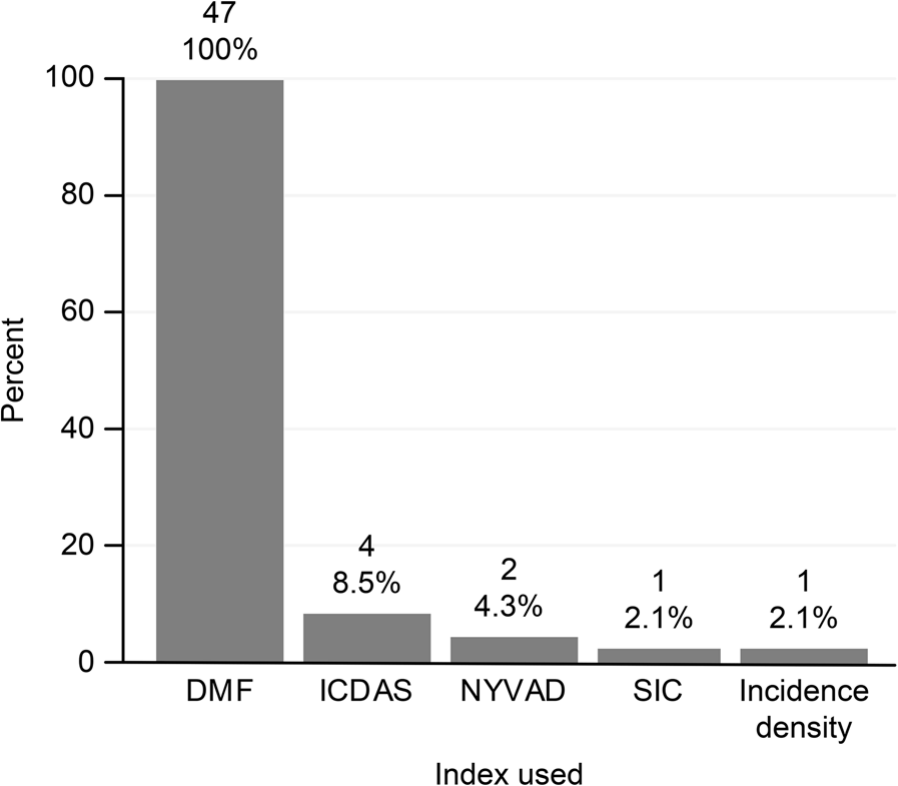
Use of caries detection methods among public oral health professors and researchers

All respondents said they had used the DMF index; of this group, 41 participants (87.2%) used only the DMF, and six used other indices (12.7%). Only the ICDAS, NYVAD, SIC, and incidence density measurements were used in practice at any time by the professionals (see Fig. 2 and Table 2).

The main reason given by respondents regarding their index of choice was the possibility of later data comparisons (66.0%), followed by the WHO and Brazilian Ministry of Health (MS) recommendations (55.3%), and because it is the most widely known index (29.8%). Ten respondents (21.3%) cited its ease of application (see Table 3).

**Table 3:** The distribution of professors and researchers by their reason for choosing an index

VARIABLES	Number	Percent
REASON FOR CHOOSING AN INDEX ^a^		
Comparability	31	66.0
WHO or MS recommended	26	55.3
Very well known	14	29.8
Easy to apply	10	21.3
ADVANTAGES OF THE DMF		
Easy to apply	19	40.4
Comparability	18	38.3
Very well known	9	19.1
Others	1	2.2
DISADVANTAGES OF THE DMF		
Does not assess enamel lesions	19	40.4
Underestimates caries prevalence	9	19.2
Mean value does not discriminate components	7	14.9
Others	12	25.5
REASONS FOR NOT USING AN INDEX		
Difficult to apply	15	31.9
Complex	13	27.7
Difficult calibration	7	14.9
Time consuming	7	14.9
Others	5	10.6
REASONS FOR PREFERRING AN INDEX		
Ease of application	2	25.5
Comparability	10	21.3
More accurately evaluates caries	7	14.9
Speed	6	12.8
Others	12	25.5
WOULD USE AN INDEX OTHER THAN THE DMF		
Yes	42	89.4
No	5	10.6

^a^ The total number of responses is greater than the number of respondents because some of the respondents provided more than one answer

According to Table 3, all participants said they had used the DMF index. The most cited advantages of this index were its ease of application (40.4%), comparability (38.3%), and the fact that it is widely known (19.1%). The most cited disadvantage of this index was that it does not allow for the detection of enamel lesions (40.4%). The second most cited disavantage was that it underestimates the prevalence of caries (19.1%), and the fact that the mean DMF value does not discriminate among decayed, missing, and filled teeth (14.9%).

As Table 3 shows, being difficult to apply (31.9%) and complex (27.7%) were the most frequently reported reasons for not using an index. Comparability (21.3%) was the second most mentioned reason to prefer an index, which was also described as the main reason for choosing one (66.0%).

In the sample analysed, 45 of the 47 respondents claimed to be dissatisfied with the DMF index (95.7%). Nevertheless, the DMF was the most widely used index and was known by all respondents. When criticisms of the indices were surveyed, respondents said that new indices should be used (36.2%), that these indices should overcome the limitations of the DMF (21.3%), and that the latter needs be replaced with other caries assessment methods (17%; see Table 4).

**Table 4:** Distribution of professors and researchers by their suggestions and most frequent index criticisms

VARIABLES	Number	Percent
SATISFIED WITH THE DMF		
Yes	2	4.3
No	45	95.7
SUGGESTIONS		
Simple and easy to understand	17	36.2
Easy to apply	9	19.1
Overcome the accommodation of using only the DMF	7	14.9
Assesses enamel lesions	6	12.8
Others	8	17.0
CRITICISMS		
New indices should be used	17	36.2
Should overcome the DMF’s limitations	10	21.3
Replace the DMF with another index	8	17.0
Research how to improve indices	7	14.9
Others	5	10.6

Five respondents stated that they would not use new indices; these participants were over 40 years of age. Two participants said that they were satisfied with the DMF, and three stated that they did not know other methods to measure caries and therefore would not use new indices.

According to the respondents’ suggestions, the methods used to assess caries must be easy to understand (36.2%) and apply (19.1%). They believe that it is necessary to overcome the accommodation of using only the DMF (14.9%) and that enamel lesions should be included (12.8%; see Table 4).

**Fig. 3:**
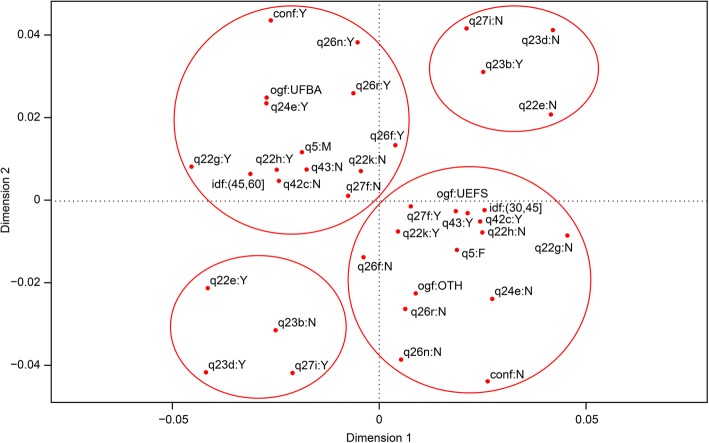
The MCA of the responses of public oral health professors and researchers. legend: ogf - place of graduation, idf – age, qusof - used an index other than DMF, q5 – sex, indf - individual knows an index other than DMF, q22g - reason for choice was WHO recommendation, q22e - reason for choosing index was comparability, q22h - reason for choice was MS recommendation, q22k - reason for choosing index was it is well known, q23b - DMF’s advantage is ease of application, q23d - DMF’s advantage is comparability, q24a - DMF’s disadvantage is not measuring enamel lesions, q24e - DMF’s disadvantage is it underestimates caries, q26f - reason for not choosing index is difficult to apply, q26n - reason for not choosing index is its complexity, q26r - reason for not choosing index is it is time consuming, q27f - prefers index due to its ease of application, q27i - prefers index due to comparability, q28 - whether would use an index other than DMF, q42c - index should be simple, q43 - whether research should be conducted to find new indices, q42m - index should be easy to apply

Figure 3 displays a graphical representation of the MCA on a two-dimensional plane. This method jointly assesses how the responses are presented, without dependency relationships or prior assumptions; similar responses are presented graphically on the opposite side to dissimilar ones.

By analysing the point projections on the axes, the responses are categorised into four different groups so that the variables belonging to each group are close together and therefore associated. The groups in opposite quadrants have large distances between their projections, thereby indicating great dissimilarity among these responses.

In the upper left quadrant group, the proximity of the points indicates associations among those who graduated from UFBA; they were older (45–60 years), male, knew indices other than the DMF, recognised the disadvantages of the DMF with regard to underestimating caries but continued to use the index because of its comparability and because it is the index recommended by the World Health Organisation (WHO) and the Brazilian Ministry of Health (MS), and would not accept using an index that was complex, difficult to apply, or time consuming.

In the lower right quadrant, the variables associated form a group composed of females who graduated from UEFS or another university; they were younger (20–45), did not know indices other than the DMF, thought new research should be conducted to find new indices to assess caries, and suggested that these new indices should be simple. They used the DMF because it is widely known; however, they did not mention that this index is recommended by the WHO or MS, and their main reason for preferring an index was its ease of application.

The lower left quadrant shows a group consisting of those mentioning comparability as an advantage of the DMF and who also cited comparability as a reason for choosing or preferring an index. In the upper right quadrant, an association was found regarding responses indicating ease of application as an advantage of the DMF, who tended not to mention comparability as a reason to choose or prefer an index, and who did not mention comparability as an advantage of the DMF.

## Discussion

According to the results presented, most people interviewed were female (70.2%), these results are in line with several studies that have shown the increasing participation of women in dental schools in Brazil [20–23].

All of the participants interviewed knew of the DMF index, but only 22 knew other caries assessment methods. Interestingly most professors taught only this index, reproducing what they had learned during their own training. Consequently, new generations of professionals will likely continue to be unaware of alternative methods.

The DMF was also the index most used by respondents, even though the vast majority of individuals surveyed claimed to be dissatisfied with it (only two people reported satisfaction). The explanation for this discrepancy lies in the reasons that led the respondents to choose an index. According to the correspondence analysis, the group who was older, male, and trained at UFBA, chose the DMF because of its comparability and because it was recommended by the WHO and MS. The younger female group who were trained at UEFS and other universities used the DMF because it was well known and easy to apply.

UFBA is the oldest university in the region, with a higher average age of the respondents, which may explain the greater concern to follow the norms of the WHO and MS, while the UEFS has a lower average age of those interviewed who were more concerned with the ease of application of the method.

Comparability was the most cited reason for choosing an index and the second most commonly reported advantage of the DMF. A concern with being able to later compare one’s findings in an oral health survey is commendable because every well-trained researcher does so. However, this preference might be establishing a vicious cycle because only the DMF index is used. The only data available for comparison are those in this index; if other methods developed to detect caries in a population are never used, then other comparisons are not possible.

Difficulties in calibration and time consumption were the reasons mentioned not to use an index, suggesting that the use of a large-scale index depends on its simplicity. This finding is in accordance with the most frequently cited reason by the group interviewed for preferring an index (i.e., its ease of application), so the respondents are concerned about using a method that is quick and easy to apply.

Ease of application was cited as the main reason for preferring an index and the most frequently cited advantage of the DMF. However, if an index is used because it is the easiest but not because it is the best, then new methods that more accurately assessing this disease will never be developed [7]. A new method to assess caries might be more difficult to use; however, its results might be better as demonstrated by other studies [4, 24]. To assess whether a change is necessary, studies that compare indices and perform cost-benefit analyses are essential.

Most respondents would use a new method to detect dental caries. It was suggested that new indices should be simple, easy to apply, and overcome the DMF’s limitations. These findings show that much of this academic community was open to accepting changes in the paradigm of how to assess caries in a population, contrary to Ismail’s [3] criticism of the dental community, for being extremely conservative and slow to accept changes.

Several interviewees noted the need to use indices that assess non-cavitated enamel lesions, which is in accordance with what many authors have made this argument in the literature [3, 25]; they believe that this change is fundamental to improve the planning of health actions based on oral health surveys.

According to Pitts [26], complex and strong barriers prevent the implementation of new caries detection methods. In the present study, the potential barriers detected, in the studied group, included a lack of knowledge of new possibilities for measuring caries in a population, the prospect of being unable to compare data after using new indices, and a belief in the possibility that new indices would be more complex and difficult to apply to measure enamel lesions. It is essential to break down these barriers and use the best tools in teaching and research because it is through caries assessment methods that the presence of this disease is assessed and strategies outlined to combat it and prevent its occurrence in a population.

Using the DMF to diagnose caries lesions leads to underestimation of caries because non-cavitated enamel lesions are ignored, thereby obstructing earlier diagnoses of the disease, which might enable planning health actions more focused on dental caries prevention.

The current study revealed some of the barriers that exist regarding the implementation of new methods, indicating the necessity of a greater discussion on the subject and showing a dissatisfaction with the current methods that are often chosen for convenience because they are easy and known. Despite the local nature of this study, it can be assumed that many other regions and cities would show similar results.

Thus far, we have not found another study that has investigated the knowledge and reasons leading to the choice of caries measurement methods in the population; as such, this information is new and should stimulate reflection on this important subject. The professionals of the area should know about the advantages and disadvantages of the various methods so that they can seek better methods to measure dental caries.

According to Ismail [3], the dental community has paid little or no attention to the complex problem of caries assessment and diagnosis. However, it is necessary to change the paradigm of caries detection levels because detecting caries at an early stage, before cavitation, can have a significant effect on the population’s oral health.

Conferences, panel discussions, and other activities should occur at universities and public oral health congresses to provide further discussion of the reasons for teaching and using a particular caries detection method to discuss the best way to assess caries in a population.

### Study limitations

This exploratory study aimed to unveil the reasons why professionals do not use new methods to measure caries. It is a local study, and therefore, its results cannot be generalised, and inferences cannot be made because the answers might differ across other cities and countries. Studies in different places should be conducted to verify whether other reasons exist for resistance to the implementation of new indices and whether the level of knowledge of the indices differs from that found in the present study.

## Conclusions

The DMF index was the best-known method and used by all respondents in teaching, research, and epidemiological caries surveys. The interviewed professionals had little knowledge of, and had seldomly used, other caries assessment methods.

Some professionals at major universities such as UFBA remain conservative because they knew of other indices but preferred to continue using the DMF because of its comparability and the fact that it is recommended by the WHO and MS. Another group composed of females who graduated from UEFS or other universities and who were younger used the DMF because it is well-known, simple, and easy to apply.

Many of the respondents demonstrated a desire for change and were critical of the DMF, although they neither knew nor used many of the current alternatives that seek to overcome the limitations of this index.

## Additional file

Additional file 1: Questionnaire about the knowledge and use of caries indices in the context of research and teaching over the last 10 years. Questionnaire applied during the interviews. (DOCX 23 kb)
