# Supplementary material for: The knowledge and use of population-based methods for caries detection
Source: BMC Oral Health. 2018 Aug 29;18:153. doi: 10.1186/s12903-018-0612-5 (PMC6116571; doi:10.1186/s12903-018-0612-5)
Supplement: Supplementary file 1 — Questionnaire about the knowledge and use of caries indices in the context of research and teaching over the last 10 years. Questionnaire applied during the interviews. (DOCX 23 kb) [file 12903_2018_612_MOESM1_ESM.docx]

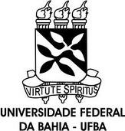


**Questionnaire about the knowledge and use of caries indices in the context of research and teaching over the last 10 years**

RESPONSIBLE: Researcher: Ana Luiza Sarno Castro RG: 3846261-30 Tel. 36711583

Advisor: Carlos Maurício Cardeal Mendes

Co-Advisor: Maria Isabel Pereira Vianna

Name ______________________________________ Birth Date: ___ / _____ / _____

Sex: (1) male (2) female

Graduation Year: ___________

Place of Work: ______________________________________________________

Professor of collective oral health?

Postgraduate with work in the area of caries indices?

If a teacher of collective oral health:

Institution where you teach:

Use of caries indices: () Teaching () Research () Extension () the main options

If you work with caries indices in teaching: What indices are covered in your theoretical classes? What indices are covered in your practicums?

Since 2005, have you participated in published studies using caries indices?

Have you performed unpublished research using caries indices?

What caries indices are you familiar with?

What caries indices did you learn during your graduate training?

What caries indices did you learn during your postgraduate training?

What caries indices have you used? Which is the most used?

What was your reason for choosing this index?

What are the advantages and disadvantages of this index?

What indices are you familiar with but have never used? Why?

What would you do if you preferred to use one index over another?

Would you use another index, different from the one you have used?

Is there any protocol or recommendation from an institution to choose a particular caries index?

Was financial influence a consideration for your choice of caries index?

Do you have any criticisms or suggestions regarding the indices that measure dental caries? Which one(s)?

Should research be performed on new indices to measure dental caries?
